# Oral and Intestinal Bacterial Substances Associated with Disease Activities in Patients with Rheumatoid Arthritis: A Cross-Sectional Clinical Study

**DOI:** 10.1155/2022/6839356

**Published:** 2022-02-18

**Authors:** Kaori Kitamura, Hiroshi Shionoya, Suguru Suzuki, Richio Fukai, Shinichi Uda, Chiyuki Abe, Hiromitsu Takemori, Keita Nishimura, Hisashi Baba, Kou Katayama, Kuniaki Terato, Takaki Waritani

**Affiliations:** ^1^Research Lab Section 5, Asama Chemical Co. Ltd., Tokyo 103-0001, Japan; ^2^Fukai Pharmacy, Asahikawa, Hokkaido 078-8243, Japan; ^3^Uda Clinic of Rheumatology, Fukuyama 721-0974, Japan; ^4^Abe Clinic Internal Medicine, Tokyo 124-0023, Japan; ^5^Aomori Prefectural Central Hospital, Aomori 030-8553, Japan; ^6^Teikyo University School of Medicine, Tokyo 173-8605, Japan; ^7^Ayumi Pharmaceutical Co. Ltd., Tokyo 104-0061, Japan; ^8^Katayama Orthopedic Rheumatology Clinic, Asahikawa, Hokkaido 078-8243, Japan; ^9^Chondrex, Inc., Woodinville, WA 98072, USA

## Abstract

Intestinal bacterial compositions of rheumatoid arthritis (RA) patients have been reported to be different from those of healthy people. Dysbiosis, imbalance of the microbiota, is widely known to cause gut barrier damage, resulting in an influx of bacteria and their substances into host bloodstreams in animal studies. However, few studies have investigated the effect of bacterial substances on the pathophysiology of RA. In this study, eighty-seven active RA patients who had inadequate responses to conventional synthetic disease-modifying antirheumatic drugs or severe comorbidities were analyzed for correlations between many factors such as disease activities, disease biomarkers, intestinal bacterial counts, fecal and serum lipopolysaccharide (LPS), LPS-binding protein (LBP), endotoxin neutralizing capacity (ENC), and serum antibacterial substance IgG and IgA antibody levels by multiple regression analysis with consideration for demographic factors such as age, sex, smoking, and methotrexate treatment. Serum LBP levels, fecal LPS levels, total bacteria counts, serum anti-LPS from *Porphyromonas gingivalis* (Pg-LPS) IgG antibody levels, and serum anti-Pg-LPS IgA antibody levels were selected for multiple regression analysis using Spearman's correlation analysis. Serum LBP levels were correlated with disease biomarker levels, such as erythrocyte sedimentation rate (*p* < 0.001), C-reactive protein (*p* < 0.001), matrix metalloproteinase-3 (*p* < 0.001), and IL-6 (*p* = 0.001), and were inversely correlated with hemoglobin (*p* = 0.005). Anti-Pg-LPS IgG antibody levels were inversely correlated with activity indices such as patient global assessments using visual analogue scale (VAS) (*p* = 0.002) and painVAS (*p* < 0.001). Total bacteria counts were correlated with ENC (*p* < 0.001), and inversely correlated with serum LPS (*p* < 0.001) and anti-Pg-LPS IgA antibody levels (*p* < 0.001). These results suggest that substances from oral and gut microbiota may influence disease activity in RA patients.

## 1. Introduction

An association between intestinal bacteria and autoimmune diseases has been suggested by a variety of studies [1–8] ever since Peltonen et al. and others reported that a vegetarian diet modulated intestinal bacterial flora and led to clinical improvement in rheumatoid arthritis (RA) patients [9–11]. Recent studies on fecal bacteria indicate that dysbiotic intestinal bacteria composition changes occur in RA patients. For example, there have been reports that RA patients showed increased levels of *Prevotella* [3, 12–15], *Staphylococcus* [12], and *Lactobacillus* [16] and decreased levels of *Bacteroides* [3, 12–14] and *Bifidobacterium* [3, 12]. Similarly, a significant compositional change in the intestinal bacteria was also observed in patients with inflammatory bowel disease (IBD) [17, 18] and spondyloarthritis [19], indicating that dysbiosis may be the fundamental disorder in a variety of autoimmune diseases.

Lipopolysaccharide (LPS), a structural component of gram-negative bacteria, is a known pyrogenic substance and is often used to develop arthritis in animal experiments [20, 21]. Elevated serum LPS levels caused by LPS absorption from the gut to the body is observed in patients with arteriosclerosis [22, 23], pediatric autoimmune neuropsychiatric disorders [24], and type 2 diabetes [25, 26], indicating that gastrointestinal barrier damage and LPS translocation into bloodstreams may play roles in the progression of various diseases.

Meanwhile, increasing evidence in clinical fields suggests a possible link between RA and periodontal infectious diseases caused by *Porphyromonas gingivalis* (*P. gingivalis*) [27–30] and *Aggregatibacter actinomycetemcomitans* [31]. It was shown that *P. gingivalis* infections significantly facilitate the development and progression of arthritis in the mouse collagen-induced arthritis (CIA) model [32–35]. Nakajima et al. [36] reported that a single oral administration of *P. gingivalis* in C57BL/6 mice had a profound impact on intestinal bacterial composition change and impaired gut mucosal barrier function, indicating that oral bacteria or their substances may be implicated in enhancing and perpetuating inflammatory arthritis. They further confirmed that *P. gingivalis* administration significantly aggravated arthritis in the mouse CIA model by modulating gut microbe populations, increasing Th17 cell populations among mesenteric lymphocytes, and concomitantly increasing serum Th17 levels [35].

In our previous study [37], we determined IgG and IgA antibody responses to LPS from *Escherichia coli* (*E. coli*-LPS), LPS from *P. gingivalis* (Pg-LPS), and peptidoglycan polysaccharide from *Streptococcus pyogenes* (PG-PS) in RA patients. We found lower IgG antibody responses to these substances to be closely correlated with RA clinical disease activity (activity indices and disease biomarkers). Based on these findings, we assume that the bacterial substances in the gastrointestinal tract may aggravate disease activity in RA patients. To confirm this hypothesis, we examined the influence of intestinal bacteria counts, bacteria-related markers such as LPS-related biomarkers, and serum IgG and IgA antibody levels against bacterial substances on RA disease activity and disease marker levels in active RA patients.

## 2. Materials and Methods

### 2.1. RA Patients and Clinical Assessment

**Figure 1:**
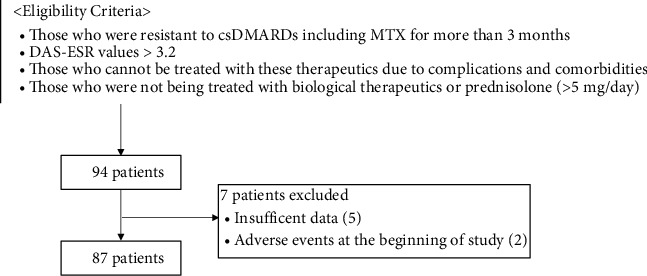
Diagram of the participant selection process.

Serum and fecal samples were obtained from ninety-four RA patients enrolled in a multicenter double blind clinical trial intended to study “the therapeutic effects and pharmacological actions of natural milk antibodies against environmental pathogens in RA,” prior to treatment (Trial Registration Number: UMIN000009492, approved by the Asahikawa Medical University Ethics Committee). To determine any beneficial effects of the milk antibody treatment on RA, the patients enrolled in this study were restricted to those with moderate-to-severe RA, who were either resistant to conventional synthetic disease-modifying antirheumatic drugs (csDMARDs) including methotrexate (MTX) for more than three months and whose disease activity score of 28 joints with erythrocyte sedimentation rate (DAS28-ESR) values remained higher than 3.2 or those who cannot be treated with these therapeutics due to complications and comorbidities. Patients who were being treated with biological therapeutics or prednisolone (>5 mg/day) were excluded. Study purposes and procedures were provided in written form, and informed consent was obtained from all patients before performing any study procedures in accordance with the Declaration of Helsinki. Patients enrolled in this study were diagnosed based on 2010 RA classification criteria by the American College of Rheumatology/European League Against Rheumatism [38]. Clinical disease activity was assessed by measuring clinical disease activity indices: tender 28 joint count (TJC), swollen 28 joint count (SJC), DAS28 with C-reactive protein (DAS28-CRP), DAS28-ESR, patient's and evaluator's global estimate for disease activity using visual analogue scale (pVAS and eVAS, respectively), VAS for pain (painVAS), modified health assessment questionnaire (mHAQ), clinical disease activity index (CDAI), and simplified disease activity index (SDAI). Patient demographic information (age, sex, disease duration, smoking, drinking, and medication) was obtained from doctor interviews. Excluding seven withdrawn patients due to insufficient data (*n* = 5) and adverse events at the beginning of study (bad feeling about taste 1, herpes zoster 1), eighty-seven out of ninety-four patients completed this study without any severe adverse effects. We employed the completed data sets from these eighty-seven patients for the analysis in this study (Figure 1).

### 2.2. Serum and Blood Samples

Serum and blood samples were obtained from individual patients before treatment and sent to a clinical laboratory to determine baseline hematological and disease marker values: ESR (mm/hr), CRP (mg/dl), rheumatoid factor (RF) (IU/ml), anti-cyclic citrullinated peptide antibody (ACPA)(U/ml), matrix metalloproteinase-3 (MMP3) (ng/ml), and hemoglobin (Hb) (g/dl) levels.

### 2.3. Fecal Samples

Fecal samples were collected by individual patients and shipped to Asama Chemical Co. Ltd. In this experiment, the water content in the fecal samples was determined to obtain an accurate count of bacteria number per gram of dry feces.

### 2.4. Reference Bacteria Strains and Culture Conditions

Five strains of reference bacteria, *E. coli* (O111:B4), *Staphylococcus aureus* (FDA209P), *Lactobacillus casei* (TISTR 390), *Bifidobacterium longum* (BB536), and *Bacteroides fragilis* (JCM 11019, NCTC 9343), were cultured in appropriate broths under anaerobic or aerobic conditions as described by Benno et al. [39] (Supplementary Table S1). Colony-forming units (CFU) were determined using colony-counting agar plates.

### 2.5. Determining Fecal Bacterial Counts by Real-Time Polymerase Chain Reaction

Bacterial DNA extraction was performed according to the method described by Matsuki et al. [40]. DNA was prepared in the same manner from the five strains of reference bacteria and used as standards. Real-time polymerase chain reaction (real-time PCR) was performed with an ABI 7300 cycler (Applied Biosystems, Tokyo, Japan) using SYBR Premix Ex Taq™ II (Tli RNase H Plus) (Takara Bio, Shiga, Japan) to quantitatively measure the amount of PCR products by fluorescence [41–45] (Supplementary Table S2).

### 2.6. Serum LPS Assay

Serum LPS was assayed by Limulus amebocyte lysate (LAL) assay using Pyrochrome with Glucashield Buffer (Seikagaku Corp., Tokyo, Japan). Serum LPS levels were expressed as pg/ml.

### 2.7. Fecal LPS Assay

The bacteria-free supernatant fraction of the fecal samples was diluted with endotoxin-free distilled water at 1 : 10^3^- to 1 : 10^6^-folds and then assayed for LPS by the LAL assay as described above, except for a 15-minute incubation time with LAL. Fecal LPS levels were expressed as *μ*g of LPS/gram of dry feces.

### 2.8. Serum Endotoxin Neutralizing Capacity Assay

Endotoxin neutralizing capacity (ENC) was measured by the method described by Bölke et al. [46]. Briefly, serum samples were diluted 10-fold with an isotonic sodium chloride solution. 5 ng of *E. coli*-LPS O111:B4 (Sigma) was incubated with 0.1 ml of the diluted serum at 24°C for 60 minutes. The active endotoxin (LPS) amount remaining in the diluted serum samples was determined by the LAL assay as described above. Serum ENC levels were expressed as ng of LPS neutralized per ml of serum.

### 2.9. Serum LPS Binding Protein Assay

Serum LPS-binding protein (LBP) levels in samples were determined by a Human LBP ELISA kit (Biometec, Germany) and expressed as *μ*g of protein/ml of serum.

### 2.10. Antibody Assay

IgG and IgA antibody responses to the three bacterial substances, ultrapure *E.coli*-LPS (O111:B4) (List Biological Laboratories, Campbell, CA), ultrapure Pg-LPS (InvivoGen, San Diego, CA), and PG-PS (Lee Laboratories, Grayson, GA), and a synthetic cyclic citrullinated peptide (Biosynthesis, Lewisville, TX) were assayed by ELISA using the ChonBlock™ buffer system (Chondrex, Inc., Woodinville, WA) [47].

### 2.11. Cytokine Assay

Serum tumor necrosis factor alpha (TNF) and interleukin-6 (IL-6) levels were assayed by Quantikine HS ELISA (R&D Systems, MIN, USA) and expressed as pg/ml.

### 2.12. Statistical Analysis

All the variables determined in this study indicated nonnormal distributions. The statistical relationships between variables were analyzed by Spearman's nonparametric rank correlation analysis (JMP10 SAS Institute Inc., Cary, NC) and expressed as Spearman's rank correlation coefficient (*ρ*). The differences in subgroups among intestinal bacterial counts and the bacteria-related markers of RA patients were analyzed by Wilcoxon signed-rank test. Multiple regression analysis was performed with serum LBP levels, fecal LPS levels, total bacteria counts, anti-Pg-LPS IgG antibody levels, and anti-Pg-LPS IgA antibody levels and the four demographic factors (age, sex, smoking, and MTX treatment) as independent variables.

## 3. Results

### 3.1. Patient Baseline Clinical Data in Eighty-Seven RA Patients

The study enrolled eighty-seven patients with moderate-to-severe RA, who were either resistant to csDMARDs including MTX for more than three months (DAS28 − ESR > 3.2) or could not be treated with these therapeutics due to complications and comorbidities. Patients' demographics were sex: 20 male and 67 female, age: 68.1 ± 0.9 (mean ± standard error (SE)), and disease duration (months): 136.0 ± 9.7. RA disease activity indices were DAS28-ESR: 4.69 ± 0.10, pVAS (mm): 41.9 ± 2.4, eVAS (mm): 42.3 ± 1.8, painVAS (mm): 46.0 ± 2.4, and mHAQ: 0.71 ± 0.07. Disease biomarkers were ESR (mm/hr): 36.9 ± 3.0, CRP (mg/dl): 1.2 ± 0.2, RF (IU/ml): 257 ± 60, ACPA (U/ml): 16.9 ± 2.6, Hb (g/dl): 12.6 ± 0.1, and MMP3 (ng/ml): 238 ± 74 (Table 1, A). Medications were MTX (7.8 ± 0.3 mg/week) in 55 patients (63.2%), and oral steroids (4.0 ± 0.2 mg/day) in 44 patients (50.6%), and so on (Table 1, B). Complications (cases) in the 87 patients included osteoporosis: 25, pulmonary interstitial disease: 16, hypertension: 13, history of cancer/benign tumor: 12, chronic pulmonary disease: 10, and so on (Table 1, C).

### 3.2. Relationship of Intestinal Bacterial Counts and Bacteria-Related Biomarkers with RA Disease Activities in Univariate Regression Analysis

**Figure 2:**
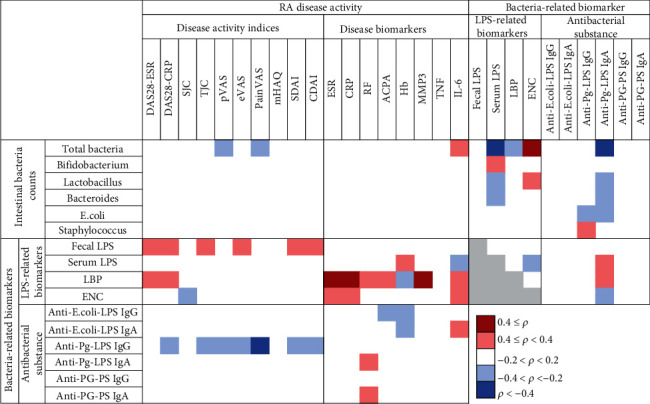
Relationship of intestinal bacterial counts and bacteria-related biomarkers with RA disease activities. The relationships between variables were expressed as Spearman's correlation coefficient (*ρ*). Light and dark red color: positive correlations, light and dark blue color: negative correlations, and gray color: not analyzed. DAS28: disease activity score with 28 joint counts; ESR: erythrocyte sedimentation rate; CRP: C-reactive protein; SJC: swollen joint count; TJC: tender joint counts; pVAS (eVAS): patient's (evaluator's) visual analogue scale; painVAS: VAS for pain; mHAQ: modified health assessment questionnaire; CDAI: clinical disease activity index; SDAI: simplified disease activity index; RF: rheumatoid factor; ACPA: anticyclic citrullinated peptide antibody; Hb: hemoglobin; MMP3: matrix metalloproteinase-3; TNF: tumor necrosis factor alpha; IL-6: interleukin-6; LPS: lipopolysaccharide; LBP: LPS-binding protein; ENC: endotoxin neutralizing capacity; Pg-LPS: LPS from Porphyromonas gingivalis; PG-PS: peptidoglycan polysaccharide.

To address the question of whether intestinal bacteria and bacterial constituents are actively involved in the pathophysiology of RA, we analyzed the relationship among intestinal bacterial counts (counts of total bacteria and five well-studied bacterial strains: *Bifidobacterium*, *Lactobacillus*, *Bacteroides*, *E. coli*, and *Staphylococcus*), bacteria-related markers (LPS-related biomarker levels and anti-bacterial substance antibody levels), and RA disease activities (activity indices and disease marker levels) using Spearman's correlation analysis, as shown in Figure 2. We noticed, especially in LPS-related biomarkers, that serum LBP levels highly correlated with disease biomarkers such as ESR (*ρ* = 0.497, *p* < 0.001), CRP (*ρ* = 0.697, *p* < 0.001), and MMP3 (*ρ* = 0.546, *p* < 0.001) (Table 2) and that fecal LPS correlated with disease activity indices such as DAS28-ESR, DAS28-CRP, SDAI, and CDAI (*p* < 0.05) (Table 3). Within the category of antibacterial substances, anti-Pg-LPS IgG antibody levels highly inversely correlated with disease activity indices such as pVAS (*ρ* = −0.376, *p* < 0.001) and painVAS (*ρ* = −0.433, *p* < 0.001) (Table 4).

### 3.3. Relationship between Individual Bacteria-Related Biomarkers in Univariate Correlation Analysis

The relationship between individual bacteria-related biomarkers was analyzed using Spearman's correlation analysis, as shown in Figure 2. It is noteworthy that total bacteria counts highly correlated with ENC (*ρ* = 0.435, *p* < 0.001) and highly inversely correlated with serum LPS (*ρ* = −0.492, *p* < 0.001) and anti-Pg-LPS IgA antibody levels (*ρ* = −0.441, *p* < 0.001) (Tables 5 and 6).

### 3.4. The Influence of Demographic Factors on Intestinal Bacterial Counts and Bacteria-Related Biomarkers

To determine how demographic factors can affect total bacterial counts, the total counts of five bacterial strains (*Bifidobacterium*, *Lactobacillus*, *Bacteroides*, *E. coli*, and *Staphylococcus*) and bacteria-related biomarkers (fecal and serum LPS, serum LBP, serum ENC, and serum IgG and IgA antibody levels against *E. coli*-LPS, Pg-LPS, and PG-PS) were determined and analyzed with respect to the following demographic factors: age, sex, disease duration, smoking, drinking, and medication (MTX, csDMARD, and steroid treatment) using Wilcoxon rank sum test. The eighty-seven patients were divided into two groups based on the unique characteristics of each factor (Supplementary Table S3 and S4). The intestinal bacterial counts and bacteria-related markers were affected by age, sex, smoking, and MTX treatment, but not affected by duration, drinking, other csDMARDs treatment, and steroid treatment. In particular, MTX treatment affected total bacteria counts (*p* = 0.004), anti-Pg-LPS IgG antibody (*p* = 0.003), and anti-Pg-LPS IgA antibody levels (*p* = 0.031) (Table 7).

### 3.5. Multiple Regression Analysis with Four Demographic Factors between Total Bacteria Counts, Serum LBP, Fecal LPS, Anti-Pg-LPS IgG Antibody, and Anti-Pg-LPS IgA Antibody Levels with RA Disease Activity, and Bacteria-Related Biomarkers

To clarify how bacterial biomarkers affect RA pathology, we focused on total bacteria counts, serum LBP levels, fecal LPS levels, and anti-Pg-LPS IgG antibody and anti-Pg-LPS IgA antibody levels among the bacteria-related markers. Their impacts were examined on RA disease activity indices and disease biomarker levels by multivariate regression analysis with consideration for demographic factors age, sex, smoking, and MTX treatment. Serum LBP levels highly correlated with disease biomarkers ESR (*p* < 0.001), CRP (*p* < 0.001), MMP3 (*p* < 0.001), and IL-6 (*p* = 0.001) and inversely correlated with Hb (*p* = 0.005) (Table 2). Although anti-Pg-LPS IgG antibody levels were significantly affected by MTX treatment (Table 6), anti-Pg-LPS IgG antibody levels inversely correlated with disease activity indices such as pVAS (*p* = 0.002) and painVAS (*p* < 0.001) (Table 4). Furthermore, total bacteria counts highly correlated with ENC levels (*p* < 0.001) and inversely correlated with serum LPS (*p* < 0.001) and anti-Pg-LPS IgA antibody levels (*p* < 0.001) (Tables 5 and 6).

## 4. Discussion

We analyzed relationships among intestinal bacteria counts, LPS-related biomarkers, serum IgG and IgA antibody levels against bacterial substances, clinical disease activity indices, and disease biomarkers, and the results showed that some bacteria-related markers correlated with disease markers of RA (Figure 2 and Tables 2–6).

Total bacteria counts were inversely correlated with serum LPS level and correlated with ENC levels (Table 5). Intestinal total bacteria counts are lower in animal models of obesity [48] and inflammatory bowel disease [49] and higher in animals treated with prebiotics [50] and probiotics [51, 52]. Hence, lower total bacteria counts may indicate dysbiosis, which can cause gut barrier destruction resulting in higher intestinal absorption of bacteria toxins such as LPS and lower ENC levels by neutralizing absorbed LPS.

Serum LBP levels were more highly associated with RA disease biomarkers (such as ESR, CRP, and MMP3) than with disease activity indices (such as DAS28 and VAS). LBP plays dual roles in the dynamics of LPS. LBP at low levels activates LPS receptors (dimerized Toll-like receptor 4: TLR4) by developing complexes with LPS and CD14, initiating the inflammation cascade and inflammatory cytokine production [53–55]. On the other hand, LBP at high levels transfers LPS to lipoproteins and chylomicrons, resulting in the clearance of LPS from the bloodstream [56–58]. This study revealed that the former LBP function may contribute to disease marker shifts by LPS, such as higher ESR, CRP, MMP3, and IL-6 levels and lower Hb levels (Table 2). Serum LBP levels at least correlated with RA disease biomarkers in this study and with RA disease activity biomarkers in another study [59], indicating that LBP may be a useful marker associated with the severity of RA, especially with accompanying gastrointestinal symptoms. However, higher LBP levels have been reported in other diseases such as Crohn's disease [60], sepsis [61], and atherosclerosis [62]. The specificity of LBP levels in RA must be evaluated with these other diseases.

The bioactivity of LPS highly depends on the origin of LPS. For example, LPS from *Bacteroidetes* antagonizes TLR4 receptor activation by *E. coli*-LPS in cell-based assays [63–65]. In addition, LPS with high bioactivity increases serum LBP levels, while LPS with low bioactivity will not [66, 67]. We speculate that LPS levels and activity may correlate with serum LBP levels and engage RA pathology. In this study, total LPS levels as derived from all gram-negative bacteria were measured by a LAL assay. Unfortunately, no method is currently available to differentiate and analyze the bioactivity of LPS in biological samples, so further studies will be necessary to confirm this speculation.

pVAS and eVAS are evaluated when achieving clinical remission [68] because pain is the most significantly obvious determinant for pVAS evaluation, while joint swelling is a more important determinant for eVAS evaluation. These discrepancies and the attempts to reconcile them can cause difficulty in achieving clinical remission for patients [69]. Zhang et al. reported that the patient's and the physician's general perceptions of disease activity are drawn from different perspectives. For example, the joint pain in RA may be due to different etiologies, including peripheral pain mechanisms with the direct activation of nociceptors, as well as sensitization of nociceptors by joint inflammation and abnormalities in the central nervous system (CNS) pain regulatory mechanisms [70]. Human nociceptor nerve terminals express several different channels and receptors related to pain sensitization, such as transient receptor potential cation channel subfamily A and V member 1 (TRPA1 and TRPV1, respectively) and TLR4. *E. coli*-LPS can bind to neuronal TLR4 to sensitize the TRPV1or directly activate TRPA1 on nociceptors [71–73]. Alternatively, Pg-LPS sensitizes TRPV1 directly [73, 74]. Therefore, different LPSs play different roles in developing pain reactions by Ca^2+^ influx through these channels when initiating a pain reaction. Pg-LPS may play roles in developing inflammation by activating TLR4 and pain by binding to TRPV1 receptors. Due to lower Pg-LPS or *P. gingivalis* absorption from the intestines or translocation in tissues, serum anti-Pg-LPS IgG antibodies can effectively neutralize active form of Pg-LPS, resulting in inactivation of the TLR4 and TRPV1 receptor. This condition may be expressed as a remission state. Instead, translocation of high levels of Pg-LPS or *P. gingivalis* that too high to be fully inactivated by existing anti-Pg-LPS antibodies, may trigger activation of the pain receptors, resulting in pain sensitization in patients. The neutralization of Pg-LPS depends on a balance of Pg-LPS levels and anti-Pg-LPS antibody levels. Therefore, we assume that anti-Pg-LPS IgG antibody levels inversely correlated with pVAS and painVAS in our study (Table 4).

In addition, joint pain is related to inflammatory cytokines such as IL-6 and granulocyte-macrophage colony-stimulating factor (GM-CSF). GM-CSF, which induces differentiation and proliferation of bone marrow progenitor cells into granulocytes and macrophages, is well known to be a multifunctional cytokine that regulates not only inflammatory responses but also pain in inflammatory diseases [75]. Locally produced GM-CSF activates sensory neurons expressing the GM-CSF receptor, transmitting painful stimuli to ascending nociceptive pathways in the spinal cord and brain [76, 77]. Viafara-García et al. reported that *P. gingivalis* or Pg-LPS treatment induced GM-CSF and angiotensin II production in coronary artery endothelial cells [78]. There might be a possibility that continual *P. gingivalis* influx from the oral cavity into the gut might be associated with patient pain due to increased GM-CSF levels. Therefore, in RA, refractory painVAS might be associated with *P. gingivalis* infection but not necessarily inflammation.

In this study, anti-Pg-LPS IgA antibody levels inversely correlated with intestinal bacteria counts such as total bacteria (*p* < 0.001), *Lactobacillus* (*p* = 0.032), and *E. coli* (*p* = 0.005) (Table 6). It has been speculated that oral bacteria colonization in the intestines is related to the pathogenesis of RA and other diseases, suggesting the existence of an oral-gut microbiome axis relationship [79]. Although we did not evaluate intestinal *P. gingivalis* counts, we speculate that the accumulation of *P. gingivalis* in the gut lumen from the oral cavity might be a cause of bacteria composition change (dysbiosis) and elevated permeability in the gut mucosal layer, leading to a submucosal translocation of bacteria and their substances into the bloodstream and initiating inflammation. Several animal studies support our hypothesis as follows: firstly, a single oral administration of *P. gingivalis* in C57BL/6 mice had a profound impact on intestinal bacterial composition and impaired gut mucosal barrier function, indicating that oral bacteria or their substances may be implicated in enhancing and perpetuating inflammatory arthritis [36]. Secondly, *P. gingivalis* inoculation downregulated intestinal gut-protective mediator and IL-10 levels as well as the expression of gut tight junction proteins in arthritic mice [80]. It has been reported that higher doses of MTX treatment can reduce bacterial numbers, especially in the *Bacteroides* group [81, 82]. With regard to the opposite effect with the MTX treatment observed in this study in Table 7 and Supplementary Table S4, we speculated that these differences may depend on the higher doses of MTX used in western countries (>15 mg/week) compared to those in Japan (~10 mg/week) as it has been reported that lower doses of MTX affect the intestinal bacteria composition and mucosal immunity differently than those with higher MTX doses [83, 84]. We assume that lower doses of MTX treatment change intestinal bacterial composition, leading to higher total bacterial counts and lower serum anti-Pg-LPS IgA antibody levels expressing immune responses against bacteria and their toxins. These changes in this study also result in lower serum LPS of a marker for inflammation and higher anti-Pg-LPS IgG antibody levels of a marker for general immune response. These results suggest that the relationship between *P*. *gingivalis* and intestinal bacteria associated with the oral-gut microbiome axis might be affected by MTX treatment, resulting in shifting RA disease activity marker levels. To clarify these marker shifts, further studies with appropriate patient groups and protocols should be considered.

**Figure 3:**
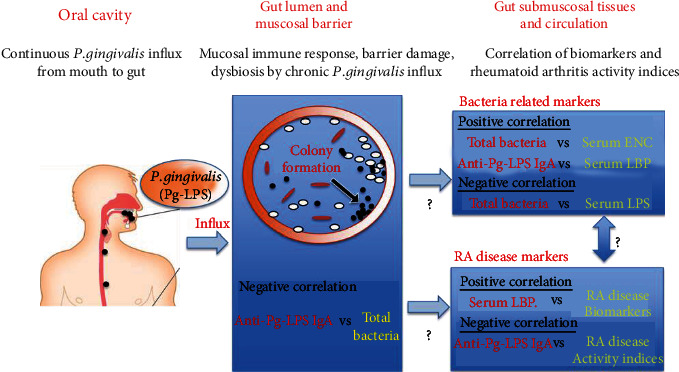
Oral-gut microbiome axis in rheumatoid arthritis.

Thus, our results suggested that substances from oral or gut microbiota may affect host immune function and can affect disease activity in RA patients (Figure 3). However, this study was a cross-sectional study with a small sample size of RA patients who had inadequate responses to csDMARDs or severe complications. To clarify the data, a larger sample size of early, untreated RA patients may be required. In a future manuscript, we will address whether the changes in the LPS-related marker levels are associated with the changes in disease markers in RA patients treated with natural milk antibodies which can modulate the intestinal bacterial composition (Katayama et al.: manuscript under preparation).

## 5. Conclusions

We confirmed that LPS-related biomarkers were correlated with not only disease activity indices but also disease biomarkers. Importantly, anti-Pg-LPS IgA antibody levels were inversely correlated with total intestinal bacteria counts and serum ENC levels, in addition to, anti-Pg-LPS IgG levels were inversely correlated with disease activity indices. These results suggest that the influx of oral *P. gingivalis* and its toxin Pg-LPS, into the gut, may change the intestinal bacterial balance and intestinal barrier function; consequently, this oral-gut microbiome axis change may aggravate disease activity in RA.

## Figures and Tables

**Table 1:** Baseline clinical data, medications, and complications.

(A) Clinical data	*N* = 87
Basic data	
Age (years)	68.1 (0.9)
Male/female	20/67
Duration (months)	136.0 (9.7)
Disease activity indices	
DAS28-ESR	4.69 (0.10)
DAS28-CRP	4.02 (0.10)
SJC	5.0 (0.3)
TJC	5.6 (0.5)
pVAS (mm)	41.9 (2.4)
eVAS (mm)	42.3 (1.8)
PainVAS (mm)	46.0 (2.4)
mHAQ	0.71 (0.07)
CDAI	19.2 (0.9)
SDAI	20.3 (0.9)
Disease biomarkers	
ESR (mm/hr)	36.9 (3.0)
CRP (mg/dl)	1.2 (0.2)
RF (IU/ml)	257 (60)
ACPA (U/ml)	16.9 (2.6)
Hb (g/dl)	12.6 (0.1)
MMP3 (ng/ml)	238 (74)
TNF (pg/ml)	2.3 (0.6)
IL-6 (pg/ml)	17.2 (2.7)
(B) Medications	*N* = 173
Methotrexate	55
Steroid	44
Bucillamine	30
Salazosulfapyridine	17
Tacrolimus	14
Leflunomide	5
Injectable gold	4
Others	4
None	1
(C) Complications	*N* = 140
Osteoporosis	25
Pulmonary interstitial diseases	16
Hypertension	13
Post cancer/benign tumor	12
Chronic pulmonary disease	10
Diabetes mellitus	9
Chronic infectious diseases	8
Rapid radiographic progression	7
Lumbar degenerative diseases	7
Post arthroplasty	6
Cardiac diseases	5
Chronic metabolic disease	5
Cervical degenerative disease	5
Others	12

DAS28: disease activity score with 28 joint counts; SJC: swollen joint counts; TJC: tender joint counts; pVAS (eVAS): patient's (evaluator's) visual analogue scale; mHAQ: modified health assessment questionnaire; CDAI: clinical disease activity index; SDAI: simplified disease activity index; ESR: erythrocyte sedimentation rate; CRP: C-reactive protein; RF: rheumatoid factor; ACPA: anticyclic citrullinated peptide antibody; Hb: hemoglobin; MMP: matrix metalloproteinase-3; TNF: tumor necrosis factor alpha; IL-6: interleukin-6. Data are shown as mean (standard error).

**Table 2:** Univariate and multivariate regression analysis between serum LBP levels and RA disease markers.

Independent variable	LBP			
Dependent variables	Univariate model		Multivariate model^a^	
	*ρ* value	*p* value	Standardized *β* (95% CI)	*p* value
DAS28-ESR	0.300	0.005^∗∗^	0.280 (0.067 : 0.493)	0.011^∗^
DAS28-CRP	0.244	0.023^∗^	0.215 (0.002 : 0.429)	0.048^∗^
ESR	0.497	<0.001^∗∗^	0.481 (0.285 : 0.676)	<0.001^∗∗^
CRP	0.697	<0.001^∗∗^	0.677 (0.517 : 0.837)	<0.001^∗∗^
RF	0.234	0.029^∗^	0.192 (-0.018 : 0.402)	0.072
ACPA	0.273	0.010^∗∗^	0.237 (0.024 : 0.449)	0.030^∗^
Hb	-0.271	0.011^∗^	-0.299 (-0.504 : -0.094)	0.005^∗∗^
MMP3	0.546	<0.001^∗∗^	0.480 (0.313 : 0.647)	<0.001^∗∗^
IL-6	0.348	0.001^∗∗^	0.316 (0.154 : 0.560)	0.001^∗∗^

DAS28: disease activity score with 28 joint counts; ESR: erythrocyte sedimentation rate; CRP: C-reactive protein; RF: rheumatoid factor; ACPA: anticyclic citrullinated peptide antibody; Hb: hemoglobin; MMP3: matrix metalloproteinase-3; IL-6: interleukin-6; *ρ*: Spearman's correlation coefficient; *β*: standardized regression coefficient; 95% CI: 95% confidence interval. Significant difference: ^∗∗^*p* < 0.01, ^∗^*p* < 0.05. ^a^Adjusted for age, sex, smoking, and methotrexate treatment.

**Table 3:** Univariate and multivariate regression analyses between fecal LPS levels and RA activity indices.

Independent variable	Fecal LPS			
Dependent variables	Univariate model		Multivariate model ^a^	
	*ρ* value	*p* value	Standardized *β* (95% CI)	*p* value
DAS28-ESR	0.237	0.027^∗^	0.230 (0.017 : 0.444)	0.035^∗^
DAS28-CRP	0.245	0.022^∗^	0.233 (0.022 : 0.443)	0.031^∗^
TJC	0.203	0.059	0.211 (0.005 : 0.416)	0.045^∗^
eVAS	0.203	0.059	0.207 (-0.003 : 0.416)	0.053
SDAI	0.233	0.030^∗^	0.217 (0.006 : 0.429)	0.044^∗^
CDAI	0.238	0.027^∗^	0.224 (0.013 : 0.435)	0.038^∗^

DAS28: disease activity score with 28 joint counts; ESR: erythrocyte sedimentation rate; CRP: C-reactive protein; SJC: swollen joint count; TJC: tender joint counts; eVAS: evaluator's visual analogue scale; SDAI: simplified disease activity index; CDAI: clinical disease activity index; LPS: lipopolysaccharide; *ρ*: Spearman's correlation coefficient; *β*: standardized regression coefficient; 95% CI: 95% confidence interval. Significant difference: ^∗^*p* < 0.05. ^a^Adjusted for age, sex, smoking, and methotrexate treatment.

**Table 4:** Univariate and multivariate regression analyses between anti-Pg-LPS IgG levels and RA disease activity indices.

Independent variable	Anti-Pg-LPS IgG			
Dependent variables	Univariate model		Multivariate model ^a^	
	*ρ* value	*p* value	Standardized *β* (95%cl)	*p* value
DAS28-CRP	-0.277	0.009^∗∗^	-0.226 (-0.448 : -0.003)	0.047^∗^
TJC	-0.218	0.043^∗^	-0.160 (-0.378 : 0.060)	0.151
pVAS	-0.376	<0.001^∗∗^	-0.353 (-0.567 : -0.137)	0.002^∗∗^
eVAS	-0.315	0.003^∗∗^	-0.271 (-0.488 : -0.053)	0.016^∗∗^
PainVAS	-0.433	<0.001^∗∗^	-0.408 (-0.614 : -0.202)	<0.001^∗∗^
SDAI	-0.308	0.004^∗∗^	-0.263 (-0.484 : -0.042)	0.021^∗^
CDAI	-0.309	0.004^∗∗^	-0.266 (-0.486 : -0.045)	0.019^∗^

DAS28: disease activity score with 28 joint counts; CRP: C-reactive protein; TJC: tender joint counts; pVAS (eVAS): patient's (evaluator's) visual analogue scale; painVAS: VAS for pain; SDAI: simplified disease activity index; CDAI: clinical disease activity index; *ρ*: Spearman's correlation coefficient; *β*: standardized regression coefficient; 95% CI: 95% confidence interval. Significant difference: ^∗∗^*p* < 0.01, ^∗^*p* < 0.05. ^a^Adjusted for age, sex, smoking, and methotrexate treatment.

**Table 5:** Univariate and multivariate regression analyses between total bacteria counts and bacterial biomarkers.

Independent variable	Total bacteria counts			
Dependent variables	Univariate model		Multivariate model^a^	
	*ρ* value	*p* value	Standardized *β* (95% CI)	*p* value
Serum LPS	-0.492	<0.001^∗∗^	-0.454 (-0.600 : -0.233)	<0.001^∗∗^
LBP	-0.242	0.024^∗^	-0.219 (-0.443 : 0.005)	0.055
ENC	0.435	<0.001^∗∗^	0.493 (0.297 : 0.689)	<0.001^∗∗^
Anti-Pg-LPS IgA	-0.441	<0.001^∗∗^	-0.402 (-0.610 : -0.194)	<0.001^∗∗^

LPS: lipopolysaccharide; LBP: LPS-binding protein; ENC: endotoxin neutralizing capacity; Pg-LPS: LPS from *Porphyromonas gingivalis*; *ρ*: Spearman's correlation coefficient; *β*: standardized regression coefficient; 95% CI: 95% confidence interval. Significant difference: ^∗∗^*p* < 0.01, ^∗^*p* < 0.05. ^a^Adjusted for age, sex, smoking, and methotrexate treatment.

**Table 6:** Univariate and multivariate regression analyses between serum anti-Pg-LPS IgA levels and bacterial biomarkers.

Independent variable	Anti-Pg-LPS IgA			
Dependent variables	Univariate model		Multivariate model^a^	
	*ρ* value	*p* value	Standardized *β* (95% CI)	*p* value
Total bacteria	-0.441	<0.001^∗∗^	-0.384 (-0.582 : -0.185)	<0.001^∗∗^
*Lactobacillus*	-0.224	0.037^∗^	-0.224 (-0.429 : -0.020)	0.032^∗^
*Bacteroides*	-0.200	0.064	-0.193 (-0.414 : 0.029)	0.088
*E. coli*	-0.260	0.015^∗^	-0.308 (-0.517 : -0.095)	0.005^∗∗^
Serum LPS	0.284	0.008^∗∗^	0.230 (0.016 : 0.406)	0.035^∗^
LBP	0.247	0.021^∗^	0.226 (0.008 : 0.444)	0.042^∗^
ENC	-0.321	0.002^∗∗^	-0.340 (-0.546 : -0.134)	0.002^∗∗^

LPS: lipopolysaccharide; LBP: LPS-binding protein; ENC: endotoxin neutralizing capacity; Pg-LPS: LPS from Porphyromonas gingivalis; *ρ*: Spearman's correlation coefficient; *β*: standardized regression coefficient; 95% CI: 95% confidence interval. Significant difference: ^∗∗^*p* < 0.01, ^∗^*p* < 0.05. ^a^Adjusted for age, sex, smoking, and methotrexate treatment.

**Table 7:** Multiple regression analysis between bacteria-related markers and demographic factors.

Dependent variable	Independent variables	Standardized *β* (95% CI)	*p* value
Total bacteria counts	Age	0.051 (-0.483 : 0.784)	0.639
	Sex^a^	0.017 (-13.61 : 15.73)	0.886
	Smoking^b^	-0.034 (-9.694 : 7.244)	0.774
	MTX	0.323 (0.631 : 3.222)	0.004∗∗

LBP	Age	0.043 (-0.532 : 0.780)	0.708
	Sex^a^	-0.129 (-23.15 : 7.216)	0.300
	Smoking^b^	0.053 (-6.872 : 10.70)	0.668
	MTX	-0.091 (-1.883 : 0.799)	0.424

Fecal LPS	Age	-0.129 (-1.038 : 0.287)	0.262
	Sex^a^	-0.105 (-21.84 : 8.836)	0.402
	Smoking^b^	-0.018 (-9.511 : 8.197)	0.883
	MTX	-0.027 (-1.515 : 1.194)	0.814

Anti-Pg-LPS IgG	Age	0.198 (-0.051 : 1.206)	0.071
	Sex^a^	0.035 (-12.39 : 16.72)	0.768
	Smoking^b^	0.098 (-4.871 : 11.94)	0.405
	MTX	0.333 (0.695 : 3.267)	0.003∗∗

Anti-Pg-LPS IgA	Age	-0.010 (-0.679 : 0.618)	0.926
	Sex^a^	0.023 (-13.59 : 16.44)	0.850
	Smoking^b^	0.068 (-6.216 : 11.12)	0.575
	MTX	-0.245 (-2.787:-0.135)	0.031∗

LBP: LPS-binding protein; Pg-LPS: LPS from *Porphyromonas gingivalis*; *ρ*: Spearman's correlation coefficient; *β*: standardized regression coefficient; 95% CI: 95% confidence interval. Significant difference: ^∗∗^*p* < 0.01, ^∗^*p* < 0.05. ^a^Men = 0, women = 1. ^b^No smoking = 1, history of smoking = 2, smoking now = 3.

## Data Availability

Authors can also make data available on request through a data access committee, institutional review board, or the authors themselves. In this case, they should name who should be contacted to request the data (e.g., the ethics or data access committee) and provide appropriate contact details. When authors have used third-party data (i.e., from another individual or source) and therefore do not own the data, this source must be credited as appropriate and details of how to access the data should be given.

## Supplementary Materials

Supplementary Materials Supplementary Table S1: reference bacterial strains and culture conditions. Supplementary Table S2: the primers used for real-time PCR. Supplementary Table S3: effects of demographic factors on bacteria counts, LPS-related biomarkers, and antibacterial substance antibody levels. Supplementary Table S4: effects of treatment on bacteria counts, LPS-related biomarkers, and anti-bacterial substance antibody levels.
