# Supplementary material for: Oral and Intestinal Bacterial Substances Associated with Disease Activities in Patients with Rheumatoid Arthritis: A Cross-Sectional Clinical Study
Source: J Immunol Res. 2022 Feb 18;2022:6839356. doi: 10.1155/2022/6839356 (PMC8881124; doi:10.1155/2022/6839356)
Supplement: Supplementary Materials — Supplementary Table S1: reference bacterial strains and culture conditions. Supplementary Table S2: the primers used for real-time PCR. Supplementary Table S3: effects of demographic factors on bacteria counts, LPS-related biomarkers, and antibacterial substance antibody levels. Supplementary Table S4: effects of treatment on bacteria counts, LPS-related biomarkers, and anti-bacterial substance antibody levels. [file 6839356.f1.pdf]

Supplementary Table S1. Reference bacterial strains and culture conditions

Strain	Source	Culture Medium	For CFU assay
Escherichia coli O111:B4	The Institute of Medical Science, The University of Tokyo	Brain heart infusion (BHI) broth (Nissui Pharmaceutical), aerobically	Pearl core plate count agar (Eiken), aerobically
Staphylococcus aureus FDA209P	Food and Drug Administration, USA	BHI broth, aerobically	Pearl core plate count agar (Eiken), aerobically
Lactobacillus casei TISTR 390	Department of Science and Technology, Thailand	MRS broth (EMD Millipore Corp.), anaerobically	MRS agar in an anaerobic chamber (Becton Dickinson and Co., Cockeysville, USA)
Bifidobacterium longum BB536	Morinaga Milk Industry, Japan	BL agar (Nissui Pharmaceutical Co., anaerobically	BL agar in an anaerobic chamber
Bacteroides fragilis JCM 11019(NCTC 9343)	Riken BioResourse Center, Japan	BHI broth, anaerobically	BHI agar in an anaerobic chamber

Supplementary Table S2. The primers used for Real-Time PCR

Bacteria		Sequence (5'-3')	PCR reaction	Product size (bp)	Reference
All Bacteria	Forward<br>Reverse	TCCTACGGGAGGCAGCAGT<br>GGACTACCAGGGTATCTAATCCTGTT	60°C-20s<br>& 72°C-20s	466	34
Bifidobacterium	Forward<br>Reverse	CTCCTGGAAACGGGTGG<br>GGTGTTCTTCCCGATATCTACA	55°C-20s<br>& 72°C-50s	549-563	35
Lactobacillus	Forward<br>Reverse	TGGAAACAGRTGCTAATACCG<br>GTCCATTGTGGAAGATTCCC	60°C-31s	231-233	36
Bacteroides	Forward<br>Reverse	ATAGCCTTTCGAAAGRAAGAT<br>CCAGTATCAACTGCAATTTTA	50°C-20s<br>& 72°C-30s	501	35
Escherichia coli	Forward<br>Reverse	GTTAATACCTTTGCTCATTGA<br>ACCAGGGTATCTAATCCTGTT	60°C-31s	340	37
Staphylococcus	Forward<br>Reverse	ACGGTCTTGCTGTCACTTATA<br>TACACATATGTTCTTCCCTAATAA	60°C- 31s	257	38

Supplementary Table S3. Effects of demographic factors on bacteria counts, LPS-related biomarkers, and anti-bacterial substance antibody levels

	Age			Sex			Duration (months)			Smoking			Drinking		
	＜65 (N=29)	≧65 (N=58)	P-value	Male (N=20)	Female (N=67)	P-value	＜36 (N=6)	≧36 (N=81)	P-value	No (N=44)	Yes [a] (N=43)	P-value	No (N=23)	Yes [b] (N=64)	P-value
Total bacteria (log/g of feces)	11.80±0.10	11.85±0.06	0.715	11.82±0.09	11.84±0.06	0.576	12.04±0.26	11.82±0.05	0.167	11.83±0.08	11.84±0.06	0.625	11.85±0.10	11.83±0.06	0.897
Bifidobacterium	9.22±0.17	9.01±0.13	0.463	9.18±0.22	9.05±0.12	0.632	9.49±0.17	9.05±0.11	0.265	9.19±0.12	8.97±0.17	0.291	9.07±0.23	9.08±0.12	0.725
Lactobacillus	8.83±0.26	9.79±0.15	0.003*	10.1±0.26	9.29±0.16	0.013*	9.68±0.48	9.46±0.15	0.719	9.45±0.22	9.50±0.18	0.875	9.88±0.24	9.33±0.17	0.101
Bacteroides	7.59±0.19	7.77±0.14	0.446	7.76±0.20	7.70±0.13	0.988	7.85±0.43	7.70±0.12	0.756	7.75±0.16	7.67±0.15	0.619	7.57±0.21	7.76±0.13	0.404
E. coli	7.73±0.28	7.82±0.18	0.631	7.69±0.32	7.82±0.17	0.655	8.46±0.53	7.74±0.15	0.203	8.02±0.21	7.55±0.20	0.094	7.77±0.30	7.80±0.17	0.915
Staphylococcus	7.32±0.17	7.62±0.13	0.172	7.46±0.21	7.54±0.12	0.848	7.29±0.37	7.54±0.11	0.558	7.61±0.15	7.43±0.15	0.476	7.39±0.24	7.57±0.11	0.421
Fecal LPS (μg/g)	29.7±10.9	20.1±3.17	0.477	39.2±15.2	18.6±2.91	0.617	16.3±3.69	23.8±4.50	0.621	20.0±4.16	26.7±7.37	0.750	26.0±11.7	22.4±3.93	0.441
Serum LPS (pg/ml)	3.90±0.22	3.93±0.14	0.698	3.80±0.24	3.95±0.13	0.648	3.76±0.48	3.93±0.12	0.756	4.03±0.17	3.80±0.16	0.248	3.72±0.20	3.99±0.14	0.353
LBP (μg/ml)	24.2±2.18	29.7±1.99	0.179	30.6±3.06	27.0±1.77	0.256	35.7±7.07	27.3±1.56	0.175	26.1±1.98	29.7±2.34	0.408	27.4±2.91	28.0±1.81	0.806
ENC (ng/ml)	14.03±0.07	14.07±0.06	0.275	13.99±0.09	14.09±0.05	0.325	14.27±0.16	14.04±0.05	0.476	14.05±0.08	14.06±0.06	0.541	14.16±0.09	14.02±0.05	0.116
Anti *E. coli*- LPS IgG (kU/ml)	4.96±0.84	7.46±0.98	0.162	5.06±1.44	7.09±0.83	0.041*	9.04±3.73	6.44±0.73	0.466	7.94±1.12	5.28±0.86	0.019*	6.15±1.01	6.79±0.91	0.973
Anti *E. coli*- LPS IgA (kU/ml)	3.92±0.81	4.61±0.64	0.390	4.31±1.06	4.40±0.58	0.576	5.92±3.01	4.27±0.50	0.874	5.07±0.71	3.67±0.71	0.012*	4.90±0.93	4.19±0.60	0.400
Anti Pg-LPS IgG (kU/ml)	82.3±29.4	63.0±12.3	0.466	44.2±8.26	77.0±16.3	0.542	44.9±16.0	71.2±13.6	0.913	69.6±19.7	69.2±16.2	0.622	56.1±12.8	74.2±16.7	0.751
Anti Pg-LPS IgA (kU/ml)	3.02±1.08	2.94±0.85	0.611	1.48±0.34	3.41±0.86	0.936	0.62±0.20	3.14±0.71	0.111	2.67±0.80	3.27±1.09	0.249	1.20±0.27	3.60±0.89	0.476
Anti PG-PS IgG (kU/ml)	207.1±33.4	239.0±19.1	0.106	221.1±31.0	230.5±20.0	0.844	264.2±78.5	225.7±17.3	0.633	220.9±23.5	236.1±24.6	0.533	231.7±29.0	227.2±20.6	0.683
Anti PG-PS IgA (kU/ml)	43.1±12.1	57.9±10.3	0.110	48.6±14.5	54.2±9.42	0.690	42.1±24.2	53.7±8.38	0.519	44.9±8.88	61.2±13.3	0.384	55.8±14.2	51.9±9.60	0.319

Patients enrolled in this study were divided into two groups depending on age, sex, disease duration, smoking, and drinking.
Effects of demographic factors on the intestinal commensal bacteria, LPS-related biomarkers, and anti bacterial antibodies levels between two groups were compared using Wilcoxon rank sum test.
Data were shown as mean±standard error and P-value. Significant difference: *P< 0.05.
LPS: Lipopolysaccharide, LBP: LPS-binding protein, ENC: endotoxin neutralizing capacity, *E. coli* -LPS: LPS from *E. coli* , Pg-LPS: LPS from *Porphyromonas gingivalis* , and PG-PS: peptidoglycan polysaccharide.
a: including patients who have a history of smoking. b: sum of patients who drink on a regular basis.

Supplementary Table S4. Effects of treatments on bacteria counts, LPS-related biomarkers, and anti-bacterial substance antibody levels

	MTX (mg/week)			MTX (mg/week) + Other csDMARDs			PSL (mg/day)		
	Non-treated (N=32)	Treated (N=55)	P-value	MTX alone (N=17)	MTX with other csDMARDs (N=38)	P-value	Non-treated (N=43)	Treated (N=44)	P-value
	MTX: 0	MTX:7.8±0.3		MTX:8.4±0.6	MTX:7.6±0.4		PSL: 0	PSL:4.0±0.2	
Total bacteria (log/g of feces)	11.65±0.09	11.94±0.06	0.004*	11.99±0.10	11.90±0.08	0.629	11.84±0.07	11.83±0.08	0.869
Bifidobacterium	8.98±0.18	9.14±0.13	0.386	9.18±0.30	9.05±0.15	0.275	9.11±0.13	9.05±0.17	0.614
Lactobacillus	9.29±0.26	9.57±0.17	0.449	9.63±0.32	9.57±0.20	0.978	9.32±0.21	9.61±0.19	0.268
Bacteroides	7.66±0.19	7.74±0.14	0.669	7.84±0.26	7.68±0.16	0.554	7.72±0.16	7.70±0.16	0.909
E. coli	7.88±0.24	7.74±0.19	0.604	7.75±0.34	7.70±0.23	0.971	7.83±0.22	7.75±0.20	0.648
Staphylococcus	7.26±0.15	7.67±0.14	0.087	7.56±0.33	7.74±0.14	0.334	7.46±0.15	7.58±0.15	0.596
Fecal LPS (μg/g)	30.8±9.99	18.9±3.18	0.993	18.6±4.02	19.1±7.88	0.472	22.6±4.45	24.0±7.13	0.986
Serum LPS (pg/ml)	4.30±0.20	3.69±0.14	0.018*	3.43±0.20	3.81±0.17	0.201	3.82±0.17	4.01±0.16	0.350
LBP (μg/ml)	30.9±2.76	26.1±1.79	0.097	26.5±3.55	25.9±2.07	0.993	28.1±2.49	27.6±1.83	0.712
ENC (ng/ml)	14.12±0.06	14.02±0.07	0.733	14.15±0.08	13.96±0.09	0.563	14.05±0.07	14.06±0.06	0.608
Anti *E. coli*- LPS IgG (kU/ml)	5.81±0.90	7.12±1.02	0.401	7.90±2.48	6.67±0.97	1.000	6.88±0.89	6.38±1.14	0.323
Anti *E. coli*- LPS IgA (kU/ml)	4.44±0.84	4.35±0.64	0.864	5.89±1.48	3.64±0.61	0.187	4.69±0.69	4.08±0.74	0.458
Anti Pg-LPS IgG (kU/ml)	61.1±21.4	74.3±15.9	0.035*	61.4±14.6	80.0±21.9	0.629	89.8±24.6	49.5±6.65	0.599
Anti Pg-LPS IgA (kU/ml)	3.77±1.31	2.50±0.73	0.005*	1.73±0.72	2.84±1.00	0.956	3.83±1.15	2.13±0.68	0.799
Anti PG-PS IgG (kU/ml)	200.3±20.9	245.6±23.8	0.269	232.7±46.2	249.4±27.6	0.387	237.6±24.8	219.3±23.2	0.494
Anti PG-PS IgA (kU/ml)	53.3±10.8	52.7±11.0	0.318	75.9±27.8	42.1±9.65	0.358	48.1±7.06	57.7±14.2	0.314

The 87 patients enrolled in this study were divided into two groups based on MTX, csDMARDs, and steroid treatment (Prednisolone: PSL). Thirty-two patients were not treated with MTX during this study period, whereas 55 patients were treated with MTX alone (N=17) or a combination of MTX and other csDMARDs (N=38). Other csDMARDs are BUC (15 cases), TAC (7), SASP (4), TAC+SASP (1), BUC + SASP (4), injectable Gold (3), Actarit (1), IGU (1), LEF (1), and injectable Gold+ BUC (1). The MTX nontreated 32 patients were treated with SASP (8), BUC (7), LEF (4), TAC (3), TAC + BUC (3), Mino (1) and AUR (1), and none (5), respectively.

Effects of treatments on the intestinal commensal bacteria, LPS-related biomarkers, and anti Pg-LPS antibody levels between the two groups were compared using Wilcoxon rank sum test.

Data were shown as mean±standard error and P-value. Significant difference: *P< 0.05.

LPS: Lipopolysaccharide, LBP: LPS-binding protein, ENC: endotoxin neutralizing capacity, *E. coli*-LPS: LPS from *E. coli,* Pg-LPS: *Porphyromonas gingivalis* LPS, PG-PS: peptidoglycan polysaccharide, MTX: methotrexate, csDMARDs: conventional synthetic disease-modifying anti-rheumatic drugs, BUC: Bucillamine, SASP: Sulfasalazine, LEF: Leflunomide, TAC: Tacrolimus, IGU: Iguratimod, Mino: Minomycin, and AUR: Auranofin.
